# Antiamnesic effect of stevioside in scopolamine-treated rats

**DOI:** 10.4103/0253-7613.66840

**Published:** 2010-06

**Authors:** Deepika Sharma, Munish Puri, Ashok K. Tiwary, Nirmal Singh, Amteshwar Singh Jaggi

**Affiliations:** 1 Fermentation and Protein Biotechnology Laboratory, Department of Biotechnology, Punjabi University, Patiala - 147 002, Punjab, India; 2 Institute of Technology Research and Innovation Centre for Biotechnology and Interdisciplinary Sciences, Institute of Technology Research and Innovation (ITRI), Deakin University, Australia; 3 Department of Pharmaceutical Sciences and Drug Research, Punjabi University, Patiala - 147 002, Punjab, India

**Keywords:** Memory, Morris water-maze, scopolamine, stevioside

## Abstract

The present study was undertaken to explore the potential of stevioside in memory dysfunction of rats. Memory impairment was produced by scopolamine (0.5 mg/kg, i.p.) in animals. Morris water maze (MWM) test was employed to assess learning and memory. Brain acetylcholinestrase enzyme (AChE) activity was measured to assess the central cholinergic activity. The levels of brain thiobarbituric acid-reactive species (TBARS) and reduced glutathione (GSH) were estimated to assess the degree of oxidative stress. Scopolamine administration induced significant impairment of learning and memory in rats, as indicated by a marked decrease in MWM performance. Scopolamine administration also produced a significant enhancement of brain AChE activity and brain oxidative stress (increase in TBARS and decrease in GSH) levels. Pretreatment of stevioside (250 mg/kg dose orally) significantly reversed scopolamine-induced learning and memory deficits along with attenuation of scopolamine-induced rise in brain AChE activity and brain oxidative stress levels. It may be concluded that stevioside exerts a memory-preservative effect in cognitive deficits of rats possibly through its multiple actions.

## Introduction

Dementia is a syndrome of progressive nature, characterized by impairment of memory and loss of intellectual ability. The dementing condition that has received the utmost attention in the past decade is Alzheimer’s disease (AD), which is the most common cause of dementia in the elderly, accounting for 60–70% of all cases. According to the World Health Organization (WHO), 5% of men and 6% of women aged above 60 years suffer from dementia of AD worldwide.[1] AD is a progressive, neurodegenerative disease characterized in the brain by the presence of senile plaques rich in insoluble aggregates of beta-amyloid and neurofibrillary tangles. Loss of cholinergic neurons in nucleus basalis magnocellularis of cortex is one of the most prominent features of AD, primarily accounting for memory loss.[2] Clinical management of dementia is still a nightmare for the neurobiologist. Thus far, cholinesterase inhibitors like donepezil, rivastigmine, galantamine, etc. are the mainstay of AD therapy. Antiinflammatory agents, antioxidants, nonsteroids and some neuroprotective agents have also been used, but with limited success.[3] Most of the currently used therapeutic interventions provide only symptomatic relief and do not halt the progression of dementia; further associated side-effects also limit their use. Hence, there is a need for an agent that will not only provide relief but will also stop the progression of the dementia.

Stevioside, the major glycoside from the leaves of *Stevia rebaudiana*, has recently gained popularity because of its sweetening nature, being about 300-times sweeter than sucrose (0.4% solution).[4] Various studies have revealed that in addition to its potential sweetening nature, it exert beneficial effects, including antihypertensive, antihyperglycemic, antihuman rotavirus, antioxidant, antiinflammatory and antitumor actions.[5] However, its antiamnesic potential remains to be explored. Therefore, the present study has been undertaken to investigate the beneficial effect of stevioside in memory deficit of rats, employing scopolamine[6] -induced amnesia as an animal model.

## Materials and Methods

### Animals

Wistar rats of either sex, weighing around 150–200 g, were employed in the present study. They were procured from the Veterinary University, Ludhiana. The rats were provided standard laboratory feed (Kisan Feeds Ltd., Chandigarh, India) and tap water. They wee exposed to an alternate light and dark cycle of 12 h and had free access to food and water. The animals were acclimatized to the laboratory conditions for at least 5 days before the behavioral test. The experimental protocol was approved by the Institutional Animal Ethics Committee (IAEC) and care of the animals was taken as per guidelines of the Committee for the Purpose of Control and Supervision of Experiments on Animals (CPCSEA), ministry of Forest and Environment, Government of India.

### Drugs and Reagents

Stevioside was purchased from Phyto-Organic, Ghaziabad, India. Folin-Ciacalteu’s phenol reagent was purchased from Merck Limited, Mumbai, India. 5,[5] dithiobis (2-nitro benzoic acid) (DTNB), reduced glutathione (GSH), bovine serum albumin (BSA) and thiobarbituric acid were obtained from Loba Chem, Mumbai, India. Scopolamine was purchased from Sigma-Aldrich, St. Louis, MO, USA. Scopolamine was dissolved in distilled water and administered intraperitoneally (*i.p*.).

### Morris-Water Maze Test (MWM)

MWM test was employed to assess learning and memory of rats.[7] The procedure described by Parle and Singh[7] was used to assess the MWM performance of the animals. Escape latency time (ELT) in seconds was recorded and day 4 ELT was taken as an index of acquisition whereas day 5 time spent in target quadrant (TSTQ) served as an index of retrieval or memory.

### Biochemical Parameters

### Collection of tissue sample

The animals were sacrificed by cervical dislocation, and the thoracic aorta and brain tissue were carefully removed. Thoracic aorta was used for endothelium-dependent and -independent relaxation, whereas brain tissue was subjected to various biochemical estimations. The removed brains were homogenized in phosphate buffer (pH 7.4, 10% w/v) using a Teflon homogenizer. The clear supernatant, obtained after centrifugation at 3000 rpm for 15 min, was used to estimate acetylcholinesterase (AChE) activity, thiobarbituric acid-reactive species (TBARS) and GSH. Blood samples for biochemical estimation were collected just before sacrificing the rats.

### Estimation of brain AChE activity

The whole-brain AChE activity was measured by the method of Ellman *et al*. with slight modification.[8] Change in absorbance per minute of the sample was read spectrophotometrically at 420 nm.

### Estimation of brain TBARS level

The whole-brain TBARS level was measured by the method of Okhawa *et al*. with slight modifications.[9] The absorbance was measured spectrophotometrically (DU 640B spectrophotometer; Beckman Coulter Inc, Kraemer Boulevard, P.O. Box 8000, Brea., 250 S, Kraemer Boulevard, P.O. Box 8000, Brea, CA, 92822-8000, USA) at 532 nm.

### Estimation of brain GSH level

The whole-brain GSH level was measured by the method of Beutler *et al*. with slight modifications.[10] The absorbance was measured spectrophotometrically at 532 nm.

### Experimental Protocol

Four groups, each comprising of seven Wistar rats, were employed in the present study.

Group I (control group): Rats were administered normal saline 30 min before acquisition trials for four consecutive days and 30 min before retrieval trial (on day 5).

Group II (scopolamine-treated control group): Rats were administered scopolamine (0.5 mg/kg, *i.p*.) 30 min before acquisition trial conducted from day 1 to day 4 and then vehicle (distilled water) only 30 min prior to retrieval trial conducted on day 5.

Group III (stevioside *per se*): Rats were administered stevioside (250 mg/kg, orally) 30 min before acquisition trial conducted from day 1 to day 4 and then vehicle (distilled water) only 30 min prior to retrieval trial conducted on day 5.

Group IV (stevioside + scopolamine-treated group): Rats were treated with stevioside (250 mg/kg, orally) 30 min before the administration of scopolamine. The remainder of the procedure was similar to that of Group II.

### Statistical Analysis

The results are expressed as mean ± standard error of means (S.E.M.). The data of behavioral results were statistically analyzed by one-way analysis of variance (ANOVA) followed by post hoc Tukey’s multiple range test using Sigma Stat Statistical software version 3.5. A *P*-value <0.05 was considered to be statistically significant.

## Results

Various pharmacological interventions employed in the present study did not show any significant mortality. Further, no significant difference was observed between the results obtained from rats of either sex.

### Effect on ELT and TSTQ Using MWM

**Figure 1:**
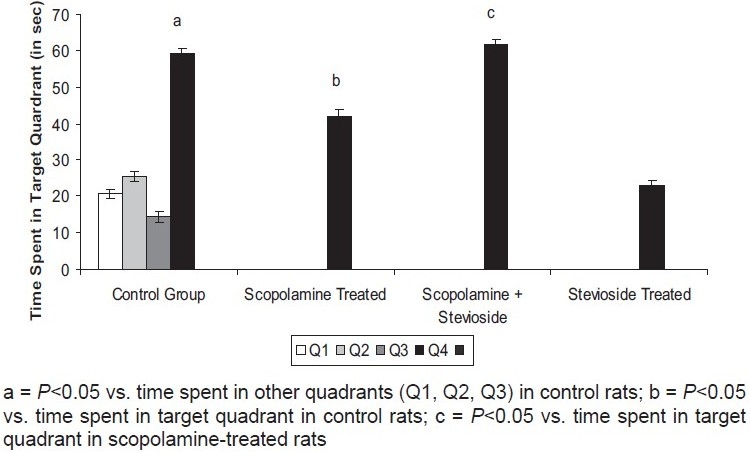
Effect of stevioside on scopolamine-induced changes in time spent in target quadrant using Morris water maze. Values are mean ± standard error of means.

Control animals showed a significant decrease in their day 4 ELT as compared to its value noted on day 1 [Table 1], reflecting acquisition (learning). Further, these mice significantly spent more time in the target quadrant (Q4) in search of missing platform as compared to the time spent in other quadrants (Q1, Q2, Q3) during retrieval trial conducted on day 5, indicating memory or retrieval [Figure 1].

**Table 1:** ELT effect of stevioside on scopolamine-induced changes in ELT using MWM

*Group*	*Dose*	*Day 1 ELT*	*Day 4 ELT*
		*(seconds)*	*(seconds)*
Control	10 mL/kg (p.o.)	89.2 ± 2.34	49.6 ± 2.17^a^
Scopolamine treated	0.5 mg/kg (i.p.)	83.8 ± 2.45	92.6 ± 4.44^b^
Scopolamine + stevioside	0.5 mg/kg (i.p.) + 250 mg/kg (orally)	86.7 ± 2.45	60.9 ± 2.45^c^
Stevioside	250 mg/kg (orally)	90.2 ± 3.62	52.5 ± 3.47

Values are mean ± S.E.M

a = *P*<0.05 vs. day 1 ELT in control rats

b = *P*<0.05 vs. day 4 ELT in control rats

c = *P*<0.05 vs. day 4 ELT in scopolamine-treated rats

Administration of scopolamine (0.5 mg/kg, *i.p*.) significantly prevented decrease in day 4 ELT of the control group [Table 1] and markedly reduced time spent in target quadrant (Q4) in search of missing platform during the retrieval trial, reflecting impairment of both learning as well as memory [Figure 1].

### Effect of Stevioside on Scopolamine-Induced Impairment of Learning and Memory Using MWM

Administration of stevioside (250 mg/kg, orally) to scopolamine-treated animals significantly attenuated day 4 rise in the ELT [Table 1] as well as day 5 decrease in TSTQ, indicating reversal of scopolamine-induced memory deficits [Figure 1].

### Effect of Stevioside on Scopolamine-Induced Changes in AChE Activity of the Brain

**Figure 2:**
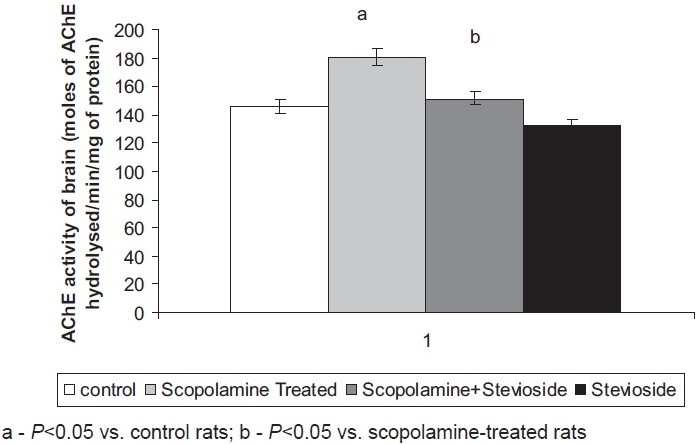
Effect of stevioside on scopolamine-induced changes in brain acetylcholinesterase (AChE) activity. Values are mean ± standard err or of means.

Scopolamine (0.5 mg/kg, *i.p*.) significantly increased the brain AChE activity when compared to control rats. Treatment with stevioside (250 mg/kg, orally) significantly attenuated the scopolamine-induced rise in brain AChE activity [Figure 2].

### Effect of Stevioside on Scopolamine-Induced Changes in the Oxidative Stress Levels of the Brain

Scopolamine (0.5 mg/kg, *i.p*.) significantly increased the brain TBARS level and reduced the brain GSH levels compared to the control group of animals, reflecting enhanced oxidative stress.

**Figure 3:**
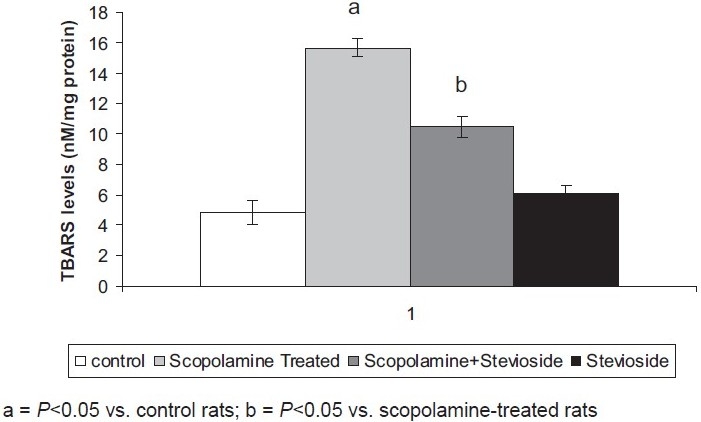
Effect of stevioside on scopolamine-induced changes in brain thiobarbituric acid-reactive species (TBARS) levels. Values are mean ± standard error of means.

**Figure 4:**
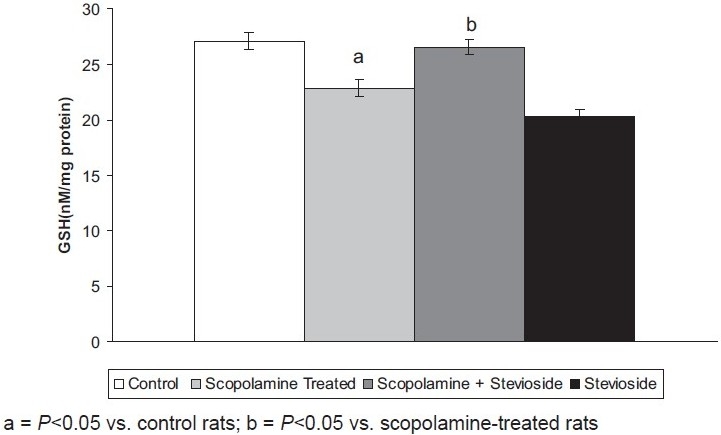
Effect of stevioside on scopolamine-induced changes in brain reduced glutathione (GSH) levels. Values are mean ± standard error of means.

Treatment with stevioside (250 mg/kg, orally) significantly abolished the scopolamine-induced rise in brain oxidative stress levels [Figures 3 and 4].

## Discussion

MWM test employed in the present study is one of the most widely accepted models to evaluate learning and memory of the animals.[7] A significant decrease in day 4 ELT of control animals during the ongoing acquisition trials denoted normal acquisition of memory and an increase in TSTQ in search of missing platform during retrieval trial indicated retrieval of memory. These results are consistent with our earlier findings.[11] In the present study, scopolamine produced impairment of acquisition and retrieval of memory, as reflected by significant increase in day 4 ELT and decrease in day 5 TSTQ, respectively. Acetylcholine (ACh) is a classic mediator of learning and memory. Drugs that reduce cholinergic function, such as muscarinic receptor antagonist scopolamine, cause profound memory impairments in animals and humans.[12] The degeneration and dysfunction of cortical cholinergic neurons is closely associated with cognitive deficits of AD.[13] These findings provide a cholinomimetic rationale for the treatment of dementia closely related to AD and support the use of animal models using muscarinic receptor antagonists.[6] This contention is further supported by our study, whereby a significant impairment of learning and memory in rats treated with scopolamine has been observed. Moreover, scopolamine treatment also produced a significant enhancement of brain AChE activity and increase in oxidative stress, as indicated by a rise in brain TBARS and reduction in GSH levels, which is in line with previous findings.[14]

In the present investigation, pretreatment of stevioside abolished scopolamine-induced memory deficits along with attenuation of scopolamine-associated increase in brain oxidative stress levels and brain AChE activity. Stevioside, being the main sweet component in the leaves of *Stevia rebaudiana Bertoni*, has recently gained much attention as a sweetener.[15] It has no caloric value and thus could be used to reduce sugar intake in diabetics, obese and patients of phenylketonuria.[16] Stevioside, in addition to its sweetening property, has also been reported to exert many other effects, including antioxidative and antiinflammatory actions.[5]

Studies in the recent past have documented the important role of sweetening agent, i.e. glucose, in learning and memory, and there is extensive evidence in the literature indicating that modest increase in circulating glucose levels enhances the formation of new memories in rodents and humans.[17] Glucose administration has been reported to enhance the memory processes by increasing hippocampal ACh synthesis and release.[18] Furthermore, extracellular brain glucose levels have been demonstrated to vary with neuronal activity, suggesting that circulating glucose may be critical in modulating neural processes important for memory functioning.[19]

Therefore, from the above findings, it is evident that stevioside abolished scopolamine-induced memory deficits. This effect may be attributed to its sweetening property. However, its potential antioxidative and antiinflammatory actions also cannot be ignored because brain oxidative stress and inflammation play a critical role in the pathobiology of dementia. Moreover, the observed anticholinesterase action of stevioside in the present investigations might also have played an important part in its antiamnestic action.

Therefore, it may be concluded that stevioside exerts its beneficial effect in scopolamine-induced memory deficits by virtue of its sweetening, antioxidative, antiinflammatory and anticholinesterase actions. Nevertheless, further studies are needed to explore the full potential of stevioside in memory deficits.
